# Simultaneous Suppression of Phonon Transport and Carrier Concentration for Efficient Rhombohedral GeTe Thermoelectric

**DOI:** 10.1002/advs.202407413

**Published:** 2024-11-17

**Authors:** Xia Qi, Te Kang, Long Yang, Xinyue Zhang, Jun Luo, Wen Li, Yanzhong Pei

**Affiliations:** ^1^ Interdisciplinary Materials Research Center School of Materials Science and Engineering Tongji University 4800 Caoan Road Shanghai 201804 China; ^2^ State Key Laboratory of High Performance Ceramics and Superfine Microstructures Shanghai Institute of Ceramics Chinese Academy of Sciences 1295 Dingxi Road Shanghai 200050 China; ^3^ University of Chinese Academy of Science 19A Yuquan Road Beijing 100049 China

**Keywords:** carrier concentration, conversion efficiency, GeTe, lattice thermal conductivity, point defect

## Abstract

Superior electronic performance due to the highly degenerated Σ valence band (*N*_v_∼12) makes rhombohedral GeTe a promising low‐temperature (<600 K) thermoelectric candidate. Minimizing lattice thermal conductivity (*κ*_L_) is an essential route for enhancing thermoelectric performance, but the temperature‐dependent *κ*_L_, corelated to *T* ^−1^, makes its reduction difficult at low temperature. In this work, a room‐temperature *κ*_L_ of ≈0.55 W m^−1^‐K^−1^, the lowest ever reported in GeTe‐based thermoelectric, is realized in (Ge_1‐_ *_y_* Sb*_y_* Te)_1‐_ *_x_* (Cu_8_GeSe_6_)*_x_*, primarily due to strong phonon scattering induced by point defects and precipitates. Simultaneously, Cu_8_GeSe_6_‐alloying effectively suppresses the precipitation of Ge, enabling the optimization of carrier concentration with the additional help of aliovalent Sb doping. As a result, an extraordinary peak *zT* of up to 2.3 and an average *zT*_avg._ of ≈1.2 within 300–625 K are achieved, leading to a conversion efficiency of ≈9% at a temperature difference of 282 K. This work robustly demonstrates its potential as a promising component in thermoelectric generator utilizing low‐grade waste heat.

## Graphical abstract

The simultaneous decreases in carrier concentration and lattice thermal conductivity by Cu_8_GeSe_6_‐alloying and Sb‐doping enable an extraordinary peak *zT* of up to 2.3 at 625 K and a superior conversion efficiency of ≈9% under a temperature difference of ≈282 K, robustly demonstrating the compound as a promising option for low‐temperature thermoelectric applications.2

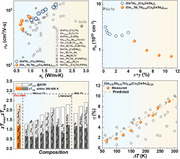

## 1 Introduction

GeTe crystallizes in a rhombohedral (r) structure below ≈700 K,^[^ ^1^ ^]^ exhibiting dominant charge conduction by the high‐energy Σ valence band that has a band degeneracy (*N*_v_) of 12. Such a large *N*_v_ results in superior electronic performance (power factor, *PF* = *S* ^2^/*ρ*, *S* and *ρ* respectively are the Seebeck coefficient and resistivity) in pristine r‐GeTe under an optimal carrier concentration (*n*).^[^ ^2^ ^]^ Furthermore, the small energy offset (Δ*E*) between Σ and low‐energy L (*N*_v_∼4) valence bands facilitates achieving an overall *N*_v_ up to 16 through the band alignment.^[^ ^3^ ^]^ Thus, a room‐temperature *PF* up to ≈26 µW/cm‐K^2^ is realized,^[^ ^4^ ^]^ strongly endorsing r‐GeTe as a promising thermoelectric candidate for low‐grade heat recovery (<600 K).

Reducing lattice thermal conductivity (*κ*_L_) is an essential avenue to improve the thermoelectric performance of GeTe. The introduction of various defects^[^ ^5^ ^]^ has been proven as an effective approach to strengthen phonon scattering and lower *κ*_L_. Note that the *κ*_L_ in general decreases with increasing temperature (*T*) following a *T* ^−1^ relationship because of the phonon scattering dominated by the Umklapp process. Although high‐temperature *κ*_L_ approaching to the amorphous limit has been frequently realized in many thermoelectric materials,^[^ ^6^ ^]^ minimization of near room‐temperature* κ*_L_ still poses a significant challenge, yet it holds great importance for low‐temperature thermoelectric applications.

Superionic semiconductors, such as Cu_2_Se^[^ ^7^ ^]^ and argyrodites (specifically Cu_8_GeSe_6_,^[^ ^8^ ^]^ Ag_8_SnSe_6_,^[^ ^9^ ^]^ and Ag_9_GaSe_6_^[^ ^10^ ^]^) have garnered intensive attention due to their inherently ultralow *κ*_L_ across the entire temperature range of interest. The origin of low *κ*_L_ is attributed to the multiple contributions of soft‐bonded Cu/Ag atoms for low sound velocity, disordered Cu/Ag atoms for strong phonon scattering, and complex crystal structures for a small population of acoustic phonons. Importantly, these compounds still maintain a high carrier mobility. These characteristics suggest a potential pathway for reducing low‐temperature *κ*_L_ of GeTe by alloying and/or compositing with these superionic compounds.^[^ ^11^ ^]^

As known, maximizing both the figure of merit (*zT*) and *PF* is only achievable within a narrow range of *n*. *n*‐optimization becomes essential for achieving the potentially highest *zT* and *PF*. Pristine GeTe has an intrinsic *n* of 10^21^ cm^−3^ due to the presence of Ge vacancies, which is considerably higher than the optimum (10^20^ cm^−3^).^[^ ^12^ ^]^ Aliovalent‐doping with Bi or Sb has been illustrated to facilitate an effective reduction in the *n*, while a doping concentration of up to 10% is usually required for optimization.^[^ ^13^ ^]^ Such high‐concentration dopants would also introduce extra carrier scattering that significantly decreases the mobility.^[^ ^14^ ^]^ Alternatively, enhancing the formation energy of Ge vacancies offers another viable approach to decrease *n* in GeTe. A noteworthy reduction in *n* has been achieved through alloying with just 2% Cu_2_Te, which leads to a negligible impact on the valence band and carrier scattering.^[^ ^15^ ^]^

All the results mentioned above motivate this work to focus on the thermoelectric performance improvement of rhombohedral GeTe by alloying Cu_8_GeSe_6_. Both lattice thermal conductivity and carrier concentration are simultaneously decreased in Cu_8_GeSe_6_‐alloyed GeTe, with relatively high carrier mobility. With the further help of Sb/Ge substitution, the lattice thermal conductivity lower than 0.55 W m^−1^‐K^−1^ across the entire temperature range and optimal carrier concentration lead to a peak *zT* of ≈2.3 and an average *zT* of ≈1.2 within 300–625 K. Eventually, a conversion efficiency of up to 9% is realized under a temperature difference of ≈282 K.

## 2 Results and Discussion

**Figure 1:**
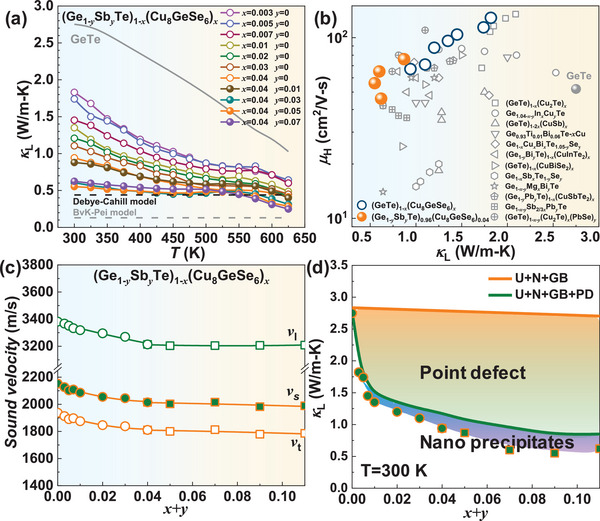
Temperature‐dependent lattice thermal conductivity (*κ*_L_) a), Hall mobility versus *κ*_L_ with a comparison to the literatures^[^ ^4^, ^6^, ^13^, ^14^, ^15^, ^18^ ^]^ b), and composition dependent sound velocities c) and *κ*_L_ predicted by the Klemens model with Born–von Karman (BvK) dispersion, considering Umklapp processes (U), normal processes (N), grain boundary scattering (GB), and substitutional point defects (PD) d) for (Ge_1‐_ *_y_* Sb*_y_* Te)_1‐_ *_x_* (Cu_8_GeSe_6_)*_x_*.

The specifics of material synthesis, characterization, transport properties, and efficiency measurements are detailed in the Supplementary Information. Cu_8_GeSe_6_‐alloying has been found to significantly reduce the *κ*_L_ of GeTe across the entire temperature range, exhibiting a progressive decline as the alloying concentration increases (**Figure** **1**a). A room‐temperature *κ*_L_ as low as ≈0.9 W m^−1^‐K^−1^ is achieved in (GeTe)_0.96_(Cu_8_GeSe_6_)_0.04_, which is further decreased to ≈0.55 W m^−1^‐K^−1^ by the Sb substitution at the Ge site, approaching to the amorphous limit according to the Debye–Cahill model (*κ*_L_^min^≈0.4 W m^−1^‐K^−1^).^[^ ^16^ ^]^ But it remains a room for further reduction according to the Born–Von Karman model taking the periodic boundary condition into account.^[^ ^17^ ^]^ It is known that the exogenous atoms would introduce additional scattering on charge carriers. A comparison of Hall carrier mobility (*µ*_H_), as depicted in Figure 1b, reveals that (Ge_0.95_Sb_0.05_Te)_0.96_(Cu_8_GeSe_6_)_0.04_ not only possesses the lowest *κ*_L_ within GeTe‐based thermoelectrics reported to date, but also accompanies a relatively high *µ*_H_. These findings suggest a possible realization of high thermoelectric performance near room‐temperature in the obtained materials.

To uncover the origin of low *κ*_L_ in (Ge_1‐_ *_y_* Sb*_y_* Te)_1‐_ *_x_* (Cu_8_GeSe_6_)*_x_*, the sound velocity of longitudinal (*v*_l_) and transverse (*v*_t_) branches, an important parameter determining *κ*_L_, is measured and shown in Figure 1c. Alloying with Cu_8_GeSe_6_ leads to a slight decrease in the sound velocities, which remain nearly constant with increasing Sb‐doping concentration. Therefore, the significant reduction in *κ*_L_ here is primarily governed by the strengthened phonon scattering. The Klemens model with Born–von Karman (BvK) dispersion^[^ ^17^, ^19^ ^]^ is employed to predict the composition‐dependent *κ*_L_ for elucidating the contributions of phonon scattering from various defects (Figure 1d). The phase composition and microstructure of the synthesized samples are initially examined.

**Figure 2:**
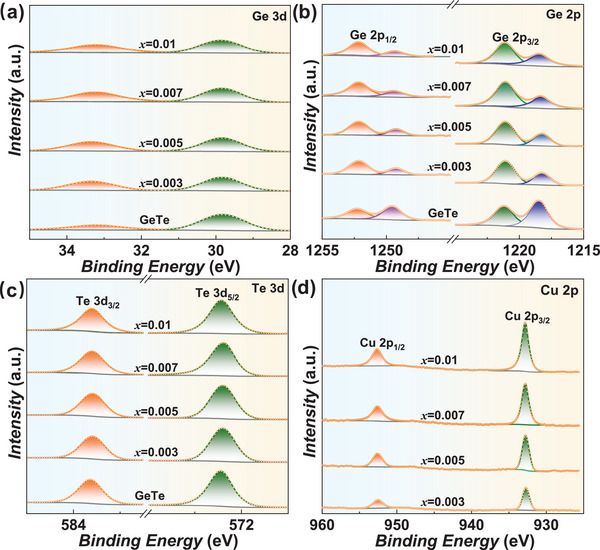
X‐ray photoelectron spectroscopy (XPS) patterns of Ge 3d a) and 2p b), Te 3d c) and Cu 2p d) for (GeTe)_1‐_ *_x_* (Cu_8_GeSe_6_)*_x_*.

**Figure 3:**
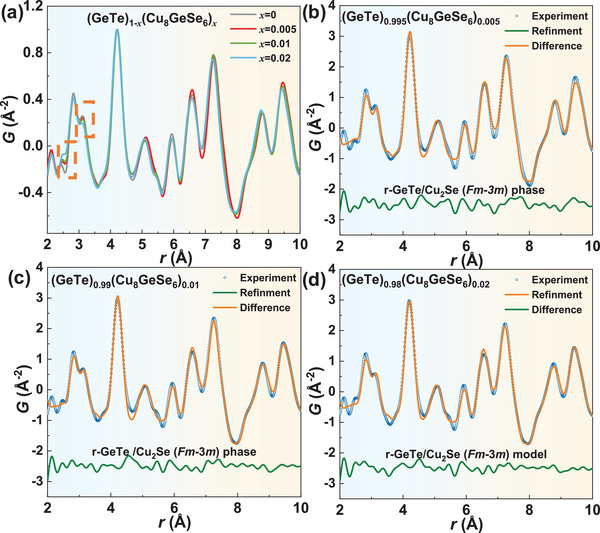
X‐ray atomic pair distribution function (PDF) datasets for (GeTe)_1‐_ *_x_* (Cu_8_GeSe_6_)*_x_* samples over the range of 2 < *r* < 10 Å a); the experimental x‐ray PDF data (blue) are fitted by the r‐GeTe phase/Cu_2_Se (*Fm*‐3*m*) model for: (GeTe)_0.995_(Cu_8_GeSe_6_)_0.005_ sample b); (GeTe)_0.99_(Cu_8_GeSe_6_)_0.01_ sample c); (GeTe)_0.98_(Cu_8_GeSe_6_)_0.02_ sample d) over the range of 2 < *r* < 10 Å. The offset is shown by the difference curve (green) below.

Room temperature powder X‐ray diffraction (XRD) patterns for (Ge_1‐_ *_y_* Sb*_y_* Te)_1‐_ *_x_* (Cu_8_GeSe_6_)*_x_* (0 ≤ *x* ≤ 0.04; 0 ≤ *y* ≤ 0.07) are shown in Figure S1 (Supporting Information). The diffraction peaks for the samples with *x* ≤ 0.02 can be well indexed to rhombohedral GeTe, indicating the formation of single‐phase. Moreover, the peaks corresponding to Cu_2_Se are detected in the samples with *x* > 0.02. To further confirm the second phase of Cu_2_Se rather than Cu_8_GeSe_6_, X‐ray photoelectron spectroscopy (XPS) and synchrotron X‐ray pair distribution function (PDF) analyses are carried out on (GeTe)_1‐_ *_x_* (Cu_8_GeSe_6_)*_x_*. As shown in **Figure** **2**, the chemical states for Ge, Cu, and Te are revealed to be +2, +1, and −2, respectively, while the Ge in Cu_8_GeSe_6_ exhibits a chemical state of +4.^[^ ^8^ ^]^ In addition, the X‐ray PDF data can be best fitted by the two‐phase model of a r‐GeTe majority phase with Cu_2_Se (*Fm*‐3*m*) (**Figures** **3** and S2 and S3, Supporting Information). More details of the PDF structural refinement results are listed in Tables S1–S3 (Supporting Information). These results conclusively identify the impurity to be Cu_2_Se.

The microstructures for (GeTe)_1_ *_‐x_* (Cu_8_GeSe_6_)*_x_* are characterized by the scanning electron microscope (SEM) and energy dispersive spectrometer (EDS) (Figures S4 and S5, Supporting Information). Precipitation of Ge occurs in pristine GeTe because of the low formation energy of Ge vacancy (Figure S4a, Supporting Information).^[^ ^2^, ^13^ ^]^ Such precipitation is effectively suppressed by Cu_8_GeSe_6_‐alloying as *x* < 0.01 (Figure S4b,c, Supporting Information), which can be rationally attributed to the increased formation energy of Ge vacancies. The analogous occurrences have also been found in Cu_2_Te‐,^[^ ^15^ ^]^ CuSbTe_2_‐,^[^ ^4a^ ^]^ PbSe‐,^[^ ^2b^ ^]^ and Sb_2_Te_3_^[^ ^14d^ ^]^‐alloyed GeTe thermoelectrics. When *x* is higher than 0.01, additional precipitates appear along the grain boundary (Figure S4d–f, Supporting Information), which are identified as Cu_2_Se according to the EDS analysis (Figure S5, Supporting Information).

**Figure 4:**
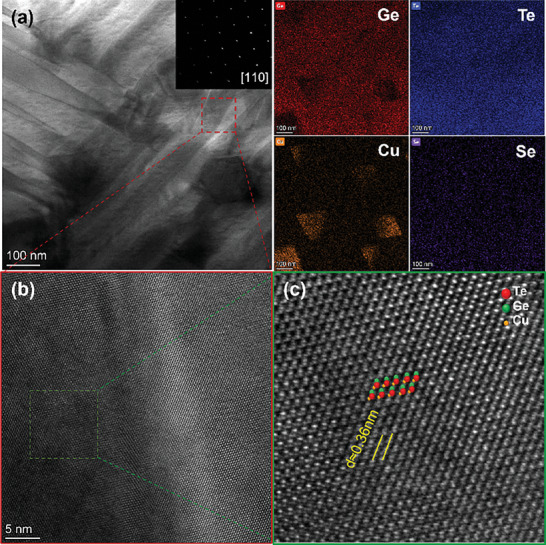
Low‐ a) and high‐resolution b,c) scanning transmission electron microscopy (STEM) images and corresponding EDS mappings for (GeTe)_0.993_(Cu_8_GeSe_6_)_0.007_.

More details of the microstructure are further characterized by the scanning transmission electron microscope (STEM), and the images are presented in **Figures** **4** and S6 and S7 (Supporting Information). Domains with hundreds of nanometers in width and several micrometers in length and Cu_2_Te particles with ≈100 nm are observed. In addition, the high‐resolution STEM image indicates that partial Cu atoms permeate the interstitial lattice of GeTe (Figure 4d). Therefore, the prediction of composition‐dependent *κ*_L_, considering only involving the contribution of point defects, is higher than the experimental results, which stems from the absence of additional domain and precipitate phonon scatterings (Figure 1d).

**Figure 5:**
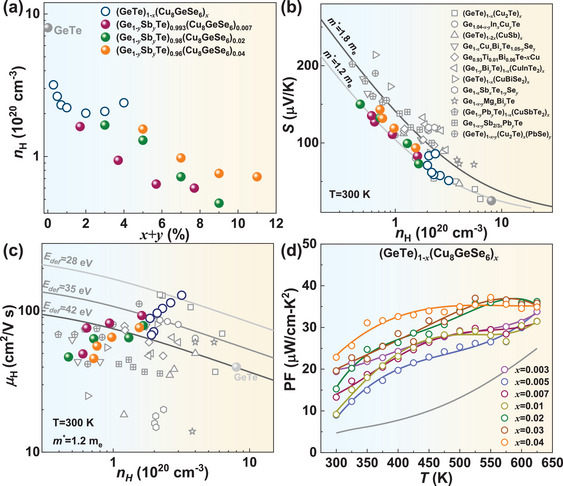
Composition‐dependent Hall carrier concentration a), *n*_H_ dependent Seebeck coefficient b) and Hall mobility (*µ*_H_, c) with a comparison to that of the literatures^[^ ^4^, ^6^, ^13^, ^14^, ^15^, ^18^ ^]^ and temperature dependent power factor (*PF*, d) for (Ge_1‐_ *_y_* Sb*_y_* Te)_1‐_ *_x_* (Cu_8_GeSe_6_)*_x_*.

In order to identify the phase transition, the differential scanning calorimetry (DSC) measurements are carried out on (Ge_1‐_ *_y_* Sb*_y_* Te)_1‐_ *_x_* (Cu_8_GeSe_6_)*_x_*, indicating the phase transition above 640 K (Figure S8,Supporting Information). Thus, the investigation of the transport properties for the samples are focused on the temperature range of 300–625 K. The transport properties for (GeTe)_1_ *_‐x_* (Cu_8_GeSe_6_)*_x_* are shown in **Figures** **5** and S9 (Supporting Information). Hall carrier concentration (*n*_H_) significantly decreases with increasing Cu_8_GeSe_6_ concentration and then remains nearly constant for *x* > 0.01 (Figures 5a and S9a, Supporting Information). This can be explained by the suppression of Ge precipitates and the saturation of Cu_8_GeSe_6_ alloying, respectively (Figures S1 and S4,Supporting Information). Moreover, Hall mobility (*µ*_H_) notably increases in the sample with *x* = 0.003, originating from the reduced concentrations of both Ge vacancies and precipitates.^[^ ^5^, ^18^ ^]^ While it decreases for *x* > 0.003 due to the additional carrier scattering by the point defects and Cu_2_Se precipitates yet remaining higher than that of pristine GeTe. Temperature‐dependent *µ*_H_ shows an unchanged mechanism of charge carrier scattering by the acoustic phonons in Cu_8_GeSe_6_‐alloyed GeTe (Figure S9b,Supporting Information).

Thus, the single parabolic band (SPB) model with the acoustic phonon scattering is utilized to predict *n*_H_‐dependent Seebeck coefficient for (GeTe)_1_ *_‐x_* (Cu_8_GeSe_6_)*_x_* (Figure 5b), which can be well described using a density‐of‐state effective mass (*m* ^*^) of ∼1.2 *m*_e_. This suggests a negligible effect of Cu_8_GeSe_6_‐alloying on the band structure of GeTe. The *µ*_H_ for (GeTe)_0.997_(Cu_8_GeSe_6_)_0.003_ can be well predicted by the SPB model with a deformation potential coefficient (*E*_def_) of ≈28 eV, smaller than that of pristine GeTe (≈42 eV), suggesting a decreased carrier scattering (Figure 5c). Temperature‐dependent resistivity and Seebeck coefficient are shown in Figure S9c,d (Supporting Information), respectively. Both increase with increasing *x* and temperature. The former arises from the decreased *n*_H_. As a result, the decreased *n*_H_ and increased *µ*_H_ lead to an effective improvement in *PF* (Figure 5d), enabling a realization of peak figure of merit (*zT*) up to 1.6 at 625 K, in addition to a decrease in *κ*_L_.

**Figure 6:**
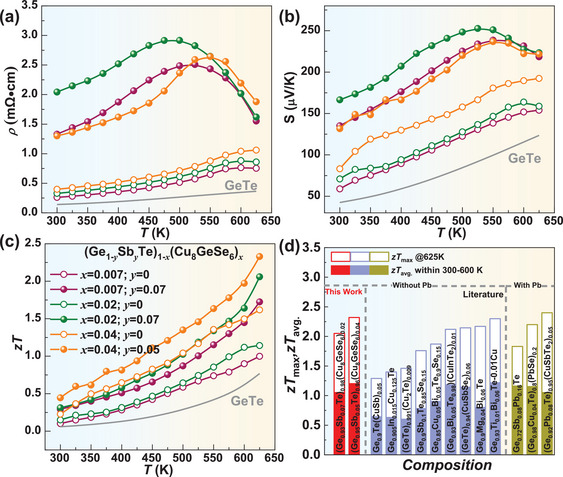
Temperature‐dependent resistivity a), Seebeck coefficient b), *zT* c), and a comparison of its peak *zT* and average *zT*_avg_. d) for (Ge_1‐_ *_y_* Sb*_y_* Te)_1‐_ *_x_* (Cu_8_GeSe_6_)*_x_* with that of the literatures.^[^ ^4^, ^6^, ^13^, ^14^, ^15^, ^18^ ^]^

It has been revealed that the Seebeck coefficient up to ≈167 µV K^−1^ is needed for maximizing the *PF*.^[^ ^20^ ^]^ Therefore, Sb is utilized as an aliovalent dopant here to further decrease the *n*_H_ of the samples with *x* = 0.007, 0.02, and 0.04, leading to an effective decrease in the *n*_H_ for (Ge_1‐_ *_y_* Sb*_y_* Te)_1‐_ *_x_* (Cu_8_GeSe_6_)*_x_*. The detailed transport properties are shown in Figures S10–S12 (Supporting Information) and **Figure** **6**. The reductions of resistivity and Seebeck coefficient at high temperatures for Sb‐doped samples are attributed to the bipolar effect. *n*_H_ dependent Seebeck coefficient is also well predicted by the SPB model using *m* ^*^ of ≈1.2 *m*_e_ (Figure 5b). These results elucidate that the increase in the Seebeck coefficient with increasing *y* stemming from the decreased *n*_H_. Thermally, Sb/Ge substitutional point defects introduce additional scattering on phonons, leading to a further reduction in *κ*_L_. Eventually, a peak *zT* up to 2.3 at 625 K and an average *zT*_ave._ of ≈1.2 within 300–625 K are achieved in (Ge_0.95_Sb_0.05_Te)_0.96_(Cu_8_GeSe_6_)_0.04_, being comparable to the highest ones reported so far^[^ ^4^, ^6^, ^13^, ^14^, ^15^, ^18^ ^]^ (Figure 6c,d). Furthermore, the Vicker hardness is found to effectively increase by Cu_8_GeSe_6_‐ and Sb‐doping, as shown in Figure S13 (Supporting Information), which is comparable to the most of the ever‐reported GeTe‐based thermoelectrics.^[^ ^4^, ^14^, ^18^, ^21^ ^]^

The legs with dimensions of 1.8 × 2.0 × 17 and 2.8 × 3.0 × 17 mm^3^ are cut for measuring the output power (*P*) and conversion efficiency (*η*) of (GeTe)_0.96_(Cu_8_GeSe_6_)_0.04_ and (Ge_0.95_Sb_0.05_Te)_0.96_(Cu_8_GeSe_6_)_0.04_ respectively, which are performed in vacuum under different temperature differences (Δ*T*). The measurement setup is shown in Figure S14 (Supporting Information), where *T*_1_ and *T*_2_ thermocouples were attached close to the hot and cold sides, respectively, for simultaneously measuring the Δ*T* and output voltage (*V*). The temperature at the cold side was fixed at 300 K using the circulating water cooling block along with a thermoelectric cooling plate. Tungsten (W) foil and copper bar were used as the electrodes to measure the output current. The foil also acts as a barrier layer to prevent the reaction of GeTe‐based thermoelectric and copper block heater. More details of the measurements are given in the Supporting Information.

**Figure 7:**
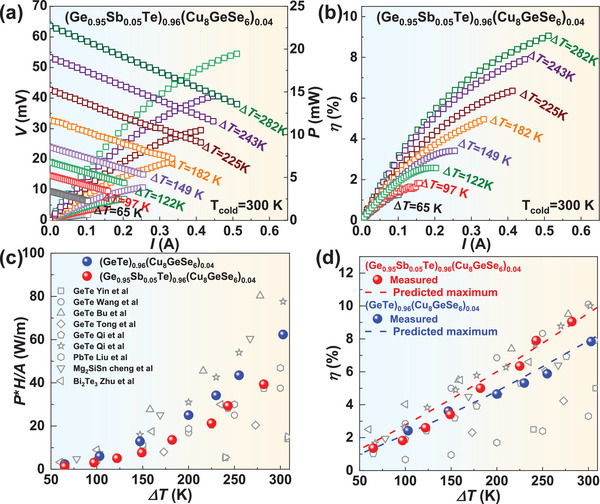
Current‐dependent output voltage and output power a) and conversion efficiency b) under different temperature differences (Δ*T*) for (Ge_0.95_Sb_0.05_Te)_0.96_(Cu_8_GeSe_6_)_0.04_. Maximum specific power density c) and maximum efficiency d) for (GeTe)_0.96_(Cu_8_GeSe_6_)_0.04_ and (Ge_0.95_Sb_0.05_Te)_0.96_(Cu_8_GeSe_6_)_0.04_, with a comparison to those of the literatures.^[^ ^4^, ^18^, ^22^ ^]^

The *V*, *P*, and *η* versus current (*I*) at different Δ*T* for (GeTe)_0.96_(Cu_8_GeSe_6_)_0.04_ and (Ge_0.95_Sb_0.05_Te)_0.96_(Cu_8_GeSe_6_)_0.04_ are shown in Figure S15a,b (Supporting Information) and **Figure** **7**a,b, respectively. The open circuit voltage (*V*_oc_) and the internal resistance (*R*_in_) are respectively determined by the intercept and the slope according to a linear fitting of *V*–*I* curves. The increases in both *V*_oc_ and *R*_in_ with increasing Δ*T* are attributed to the increased Seebeck coefficient and resistivity for thermoelectric materials at elevated temperature. The experimental *V*_oc_ and *R*_in_ agree well with the prediction using the temperature‐dependent Seebeck coefficient and resistivity (Figure S15c,d, Supporting Information), suggesting the thermal stability of the materials across the measured temperature range.

The *P* and *η* are primarily determined by the *PF* and *zT*_avg._ of thermoelectric materials, respectively. A maximal *P*_max_ of ≈20 mW is achieved in (Ge_0.95_Sb_0.05_Te)_0.96_(Cu_8_GeSe_6_)_0.04_ at Δ*T* of 282 K (Figure 7a), which corresponds to a specific power density (*P*_max_ *H*/*A*, *H* is the leg height, *A* is the sectional area of thermoelectric material) of ≈40 W m^−1^. The heat flow at different Δ*T* is shown in Figure S16 (Supporting Information). Eventually, a maximal *η*_max_ of ≈9% is realized at Δ*T* of 282 K (Figure 7b). The obtained specific power density (Figure 7c) and *η*_max_ (Figure 7d) in this work are comparable to those of GeTe‐based thermoelectric single‐legs.

## 3 Summary

Significant reductions in lattice thermal conductivity and carrier concentration of GeTe are simultaneously achieved by Cu_8_GeSe_6_‐alloying, stemming from the strong phonon scattering by point defects and precipitates and efficient suppression of Ge precipitates. With the help of Sb‐doping for further optimizing lattice thermal conductivity and carrier concentration, the peak *zT* of ≈2.3 and average *zT*_avg._ of 1.2 within 300–625 K are realized for (Ge_0.95_Sb_0.05_Te)_0.96_(Cu_8_GeSe_6_)_0.04_. The single‐leg device with a conversion efficiency of ≈9% under a temperature difference of ≈282 K, demonstrates the material as a promising option for low‐temperature thermoelectric applications.

## 4 Experimental Section

### Synthesis

Polycrystalline (Ge_1‐y_Sb_y_Te)_1‐_ *_x_* (Cu_8_GeSe_6_)*_x_* (0 ≤ x ≤ 0.04, 0.01 ≤ y ≤ 0.07) samples were synthesized by sealing the stoichiometric amounts of high purity elements (≥99.99%) in vacuum quartz ampoules, which are melted at 1270 K for 6 h and quenched in cold water, and then annealed at 873 K for 3 days. The obtained ingots were ground into fine powder for hot press and X‐ray diffraction (XRD) analysis. The dense pellets (>98% theoretical density) with ≈1.5 mm in thickness and ≈12 mm in diameter were obtained by an induction heating hot press system at 723 K under 75 MPa for 40 min.

### Characterizations and Measurements

The microstructure was characterized by a scanning electron microscope (SEM) and a scanning transmission electron microscope (STEM) equipped with an energy‐dispersive spectrometer (EDS). A Differential Scanning Calorimetry apparatus (DSC) was used to check the phase transition of the samples. Electrical properties of Seebeck coefficient (*S*), resistivity (*ρ*), and Hall coefficient (*R*_H_) were simultaneously measured in the temperature range of 300–625 K under a helium atmosphere. Two K‐type thermocouples were embedded at two sides along the radial direction of the pellets for measuring both the temperature difference and thermopower. The Seebeck coefficient was obtained from the slope of the thermopower versus temperature differences within 0–5 K. The *ρ* and *R*_H_ were measured using the van der Pauw technique under a magnetic field of 2.0 T. Thermal diffusivity (*D*) was measured by the laser flash technique. Thermal conductivity (*κ*) was estimated via *κ = dC*_p_ *D*, where *d* is the mass density measured using the mass and geometric volume of the pellets and *C*_p_ is the heat capacity estimated by the Dulong–Petit approximation with an assumption of temperature‐independent. The measurement uncertainty of *S*, *ρ*, *R*_H_, and *κ* were ≈5%.

## Conflict of Interest

The authors declare no conflict of interest.

## Supporting information



Supporting Information

## Data Availability

The data that support the findings of this study are available from the corresponding author upon reasonable request.
