# Supplementary material for: Simultaneous Suppression of Phonon Transport and Carrier Concentration for Efficient Rhombohedral GeTe Thermoelectric
Source: Adv Sci (Weinh). 2024 Nov 17;11(47):2407413. doi: 10.1002/advs.202407413 (PMC11653633; doi:10.1002/advs.202407413)
Supplement: Supplementary file 1 — Supporting Information [file ADVS-11-2407413-s001.docx]

**Supporting Information**

**Simultaneous suppression of phonon transport and carrier concentration for efficient rhombohedral GeTe thermoelectric**

Xia Qi^1,2,3,#^, Te Kang^1,#^, Long Yang^1^, Xinyue Zhang^1^, Jun Luo^1^, Wen Li^1,*^ and Yanzhong Pei^1,*^

^1^ Interdisciplinary Materials Research Center, School of Materials Science and Engineering, Tongji University, 4800 Caoan Road, Shanghai, 201804, China

^2^ State Key Laboratory of High Performance Ceramics and Superfine Mic rostructures, Shanghai Institute of Ceramics, Chinese Academy of Sciences, 1295 Dingxi Road, Shanghai, 200050, China

^3^ University of Chinese Academy of Science, 19A Yuquan Road, Beijing, 100049, China

^#^These authors contributed equally.

^*^E-mails: [liwen@tongji.edu.cn](mailto:liwen@tongji.edu.cn); [yanzhong@tongji.edu.cn](mailto:yanzhong@tongji.edu.cn)

**SPB model calculates**

The transport properties are predicted by a single parabolic band (SPB) model using the following equations^1^:

The Hall carrier concentration, $n_{H}=\frac{1}{eR_{H}}=\frac{({2m^{*}k_{B}T)}^{3/2}}{3\pi^{2}\hbar^{3}}\frac{\left( r+\frac{3}{2} \right)^{2}F_{({r+\frac{1}{2})}^{2}}(\eta)}{\left( 2r+\frac{3}{2} \right)F_{(2r+\frac{1}{2})}(\eta)}$

Where *F*_j_(η) is the Fermi integral, $F_{j}\left( \eta\right)=\int_{0}^{\infty} \frac{\xi^{j}d\xi}{1+exp(\xi-\eta)}$

The Seebeck coefficient, $S\left( \eta\right)=\frac{k_{B}}{e}[\frac{\left( r+\frac{5}{2} \right)F_{\left( r+\frac{3}{2} \right)}\left( \eta\right)}{\left( r+\frac{3}{2} \right)F_{\left( r+\frac{1}{2} \right)}\left( \eta\right)}-\eta]$

The carrier mobility, $\mu_{H}=\mu_{0}\frac{\left( 2r+\frac{1}{2} \right)F_{(2r-\frac{1}{2})}}{\left( 1+r \right)F_{r}}$

Where *k*_B_ is the Boltzmann constant, *ħ* is the reduced Planck constant, *m** is the density of states (DOS) effective mass, and *η* is the reduced Fermi level. When charge carriers are scattered by acoustic phonons, *r*=-1/2.

s

**PDF measurements**

The local structure was studied using the atomic pair distribution function (PDF) technique. The atomic PDF gives the interatomic distance distribution, i.e., the probability of finding atomic pairs of distance *r* apart^2^. The experimental PDF, denoted *G(r)*, is the truncated Fourier transform of the total scattering structure function.

$$\begin{aligned} \text{G }\left( \text{r} \right)\text{=}\frac{2}{\text{π}}\int_{\text{Q}_{\text{min}}}^{\text{Q}_{\text{max}}} \text{Q}\text{[}\text{S}\text{(}\text{Q}\text{)-1}\text{]}\sin\left( \text{Qr} \right)\text{dQ}\text{ (1) } \end{aligned}$$

where *Q* is the magnitude of the scattering momentum transfer. The total scattering structure function, *S(Q)*, is extracted from the Bragg and diffuse components of X-ray powder diffraction intensity. For elastic scattering, *Q*=4πsin*θ*/*λ*, where *λ* is the scattering wavelength and 2*θ* is the scattering angle. In practice, values of *Q*_min_ and *Q*_max_ are determined by the experimental setup and is often reduced below the experimental maximum to eliminate noisy data from PDF since the signal to noise ratio becomes unfavorable in the high-*Q* region^3, 4^.

The synchrotron X-ray total scattering measurements were carried out at the BL12SW beamline at Shanghai Synchrotron Radiation Facility (SSRF) using the rapid acquisition PDF method (RAPDF)^4^. The (GeTe)_1-_*_x_*(Cu_8_GeSe_6_)*_x_* (*x*=0, 0.005, 0.01 and 0.02) powder samples were loaded in a 1-mm-diameter polymide capillaries and measured at room temperature. The experimental setup was calibrated by measuring the crystalline CeO_2_ powder as a standard material. A two-dimensional large area flat panel detector was mounted behind the sample perpendicular to the primary beam path with a sample-to-detector distance of 0.4394 mm. The incident X-ray wavelength was 0.1225 Å. The PDF instrument resolution parameters *Q*_damp_ and *Q*_broad_^5, 6^ were determined as *Q*_damp_=0.02522 Å^-1^ and *Q*_broad_=0.02367 Å^-1^ by fitting the X-ray PDF from CeO_2_ standard material collected under the same experimental conditions, which were fixed when refining the sample data.

The detector total exposure time was 300 s for each sample for sufficient counting statistics on the data. The collected data frame was corrected for polarization effects, and masked to remove outlier pixels before being integrated along arcs of constant *Q*, where *Q*=4πsin*θ*/*λ* is the magnitude transfer on scattering, to produce 1D powder diffraction patterns using the pyFAI program^7^. Standardized corrections and normalizations were then applied to the data to obtain the total scattering structure function, *F(Q)*, which was Fourier transformed to obtain the PDF using PDFgetX3^8^. The minimum and maximum ranges of data used in the Fourier transform were chosen to be respectively *Q*_min_=0.5110 Å^-1^and *Q*_max_=21.20 Å^-1^, so as to give the best trade-off between statistical noise and real-space resolution.

The PDF modeling program PDF was used for local structure refinements^5^. In these refinements, *U_iso_* (Å^2^) is the isotropic atomic displacement parameter (ADP), and the ADPs of the same type of atoms are constrained to be the same; δ_2_ (Å^2^) is a parameter that describes correlated atomic motions^9^; the PDF instrument parameters *Q*_damp_ and *Q*_broad_ determined by fitting the PDF from the well-crystallized standard sample under the same experimental conditions were fixed in the structural refinements of the sample datasets.

**Device fabrication and efficiency measurements**

The legs with dimensions of 1.8×2.0×17 and 2.8×3.0×17 mm^3^ were cut from the ingots for devices test, which were loaded between the heater and the heat-flow meter using InGa liquid alloy. Two K-type thermocouples (*T*_1_ and *T*_2_) were adhered at two sides of the leg using silver paste for measuring both the temperature difference and output voltage. The copper bar (cross-sectional area of 3×3 mm^2^) was used as a heat-flow meter and two K-type thermocouples with a small diameter of 0.06 mm were embedded for determining the temperature difference (*T*_3_: hot side; *T*_4_: cold side). The output power is obtained via *P*=*IV*, where *I* is the current and *V* is the output voltage. The maximum output power can be obtained by changing the load resistance and measuring the corresponding *I* and *V*.

The heat flow is related to the temperature difference, the dimension and the thermal conductivity of copper (heat-flow meter) according to:

$Q=\frac{\kappa_{C\text{u}}A_{Cu}}{L_{Cu}}\Delta T_{Cu}$ (2)

where *Q*, *A*_Cu_, *L*_Cu_, Δ*T*_Cu_=*T*_3_-*T*_4_ and *κ*_Cu_ are the heat flow, the cross-section area of the heat-flow meter, the distance between the thermocouples, the temperature difference and the thermal conductivity of copper, respectively. The average *κ*_Cu_ of ~386 W/m-K is used for determining the heat flow^10^.

The conversion efficiency (*η*) is given by

$\eta=\frac{P}{P+Q}$ (3)

The maximum conversion efficiency (*η*_max_) can be obtained by varying the load resistance, and measuring the corresponding output power and heat flow. To minimize the system error, each parameter (including temperature, voltage and current) was measured 30 times for averaging.

**Prediction of device properties according to the measured properties of the material:**

The internal resistance (*R*_in_) of the single-leg device at different temperature (*T*) is estimated by:

$R_{in}=\frac{l}{A}\sum_{i=1}^{7} \frac{\int_{T_{i}}^{T_{i+1}} \rho_{i}(T_{i})dT}{T_{i+1}-T_{i}}$ (4)

where *l* is the height and *A* is the cross-sectional area of the single leg, *r_i_* (*T_i_*) is the resistivity of different parts of the single leg.

The open-circuit voltage (*V*_oc_) of the single-leg device is estimated by:

$V_{oc}=\sum_{i=1}^{7} \int_{T_{i}}^{T_{i+1}} S_{i}(T_{i})dT$ (5)

where *S_i_*(*T_i_*) is the Seebeck coefficient of different parts of the single leg.

The theoretical conversion efficiency (*η*) of the single-leg device is estimated by:

$\eta=\frac{T_{h}-T_{c}}{T_{h}}\frac{\sqrt{1+Z\overline{T}}-1}{\sqrt{1+Z\overline{T}}+\frac{T_{c}}{T_{h}}}$ (6)

where $Z\overline{T}=\frac{\sum_{i=1}^{7} \int_{T_{i}}^{T_{i+1}} (ZT)_{i}dT}{T_{h}-T_{c}}$, (*ZT*)*_i_* is the figure of merit of different parts of the leg.

Fig. S1. Room temperature powder XRD patterns for (Ge_1-_*_y_*Sb*_y_*Te)_1-_*_x_*(Cu_8_GeSe_6_)*_x_* alloys (0≤*x*≤0.04; 0≤*y*≤0.07)).

Table S1. The PDF structural refinement results for (GeTe)_1-_*_x_*(Cu_8_GeSe_6_)*_x_* fitted by the r-GeTe phase model over the range of 2<*r*<10 Å and 10<*r*<40 Å. ADP is the isotropic atomic displacement parameter.

| Range |  | *x*=0 | *x*=0.005 | *x*=0.01 | *x*=0.02 |
| --- | --- | --- | --- | --- | --- |
| 2 ~ 10 Å | lattice parameter (Å) | a=b=4.150  c=10.670 | a=b=4.161  c=10.684 | a=b=4.153  c=10.679 | a=b=4.139  c=10.637 |
|  | ADP Ge (Å^2^) | 0.0173 | 0.0149 | 0.0180 | 0.0174 |
|  | ADP Te (Å^2^) | 0.00743 | 0.00683 | 0.00733 | 0.00761 |
|  | R_w_ | 0.115 | 0.158 | 0.129 | 0.134 |
| 10 ~ 40 Å | lattice parameter (Å) | a=b=4.153  c=10.656 | a=b=4.165  c=10.666 | a=b=4.159  c=10.653 | a=b=4.144  c=10.623 |
|  | ADP Ge (Å^2^) | 0.0127 | 0.0121 | 0.0147 | 0.0134 |
|  | ADP Te (Å^2^) | 0.00808 | 0.00836 | 0.00886 | 0.00881 |
|  | R_w_ | 0.114 | 0.210 | 0.162 | 0.141 |

Table S2. The PDF structural refinement results for (GeTe)_1-_*_x_*(Cu_8_GeSe_6_)*_x_* fitted by the r-GeTe phase/Cu_2_Se (*Fm*-3*m*) model over the range of 2<*r*<10 Å. ADP is the isotropic atomic displacement parameter.

| Sample | r-GeTe phase/Cu_2_Se (*Fm*-3*m*) model | Lattice (Å) | ADP (Å^2^) | R_w_ | Mass ratio |
| --- | --- | --- | --- | --- | --- |
| (GeTe)_0.995_(Cu_8_GeSe_6_)_0.005_ | GeTe | a=b=4.168  c=10.684 | Ge:0.0142  Te:0.00760 | 0.140 | 0.890:0.110 (0.052) |
|  | Cu_2_Se (*Fm*-3*m*) | a=5.888 | Cu:0.0180  Se:0.00362 |  |  |
| (GeTe)_0.99_(Cu_8_GeSe_6_)_0.01_ | GeTe | a=b=4.155  c=10.682 | Ge:0.0192  Te:0.00800 | 0.120 | 0.950:0.050 (0.052) |
|  | Cu_2_Se (*Fm*-3*m*) | a=5.907 | Cu:0.00621  Se:0.00478 |  |  |
| (GeTe)_0.98_(Cu_8_GeSe_6_)_0.02_ | GeTe | a=b=4.142  c=10.636 | Ge:0.0176  Te:0.00777 | 0.120 | 0.903:0.097 (0.051) |
|  | Cu_2_Se (*Fm*-3*m*) | a=5.866 | Cu:0.00853  Se:0.0240 |  |  |

Fig. S2. X-ray PDF data for GeTe (a), (GeTe)_0.995_(Cu_8_GeSe_6_)_0.005_ (b), (GeTe)_0.99_(Cu_8_GeSe_6_)_0.01_ (c) and (GeTe)_0.98_(Cu_8_GeSe_6_)_0.02_ (d) fitted by the r-GeTe phase model over the range of 2<*r*<10 Å and 10<*r*<40 Å. The offset is shown by the difference curves (green) below.

Table S3. The PDF structural refinement results for (GeTe)_1-_*_x_*(Cu_8_GeSe_6_)*_x_* fitted by the r-GeTe phase/Cu_2_Se (*R-*3*m*) and r-GeTe phase/Cu_8_GeSe_6_ (*P*6_3_*cm*) models over the range of 2<*r*<10 Å. ADP is the isotropic atomic displacement parameter.

| Sample | Phase | Lattice (Å) | ADP (Å^2^) | R_w_ | Mass ratio |
| --- | --- | --- | --- | --- | --- |
| (GeTe)_0.995_(Cu_8_GeSe_6_)_0.005_ | GeTe | a=b=4.160  c=10.680 | Ge:0.0141  Te:0.00657 | 0.153 | 0.961:0.039(0.062) |
|  | Cu_2_Se(*R-*3*m*) | a=b=4.419  c=20.940 | Cu:0.00587  Se:0.0264 |  |  |
| (GeTe)_0.995_(Cu_8_GeSe_6_)_0.005_ | GeTe | a=b=4.161  c=10.684 | Ge:0.0162  Te:0.00662 | 0.158 | 1.00:0 |
|  | Cu_8_GeSe_6_  (*P*6_3_*cm*) | a=b=7.316  c=11.768 | Cu:0.00423  Se:0.00547 |  |  |
| (GeTe)_0.99_(Cu_8_GeSe_6_)_0.01_ | GeTe | a=b=4.152  c=10.678 | Ge:0.0168  Te:0.00707 | 0.124 | 0.955:0.045(0.062) |
|  | Cu_2_Se(*R-*3*m*) | a=b=4.246  c=20.823 | Cu:0.0297  Se:0.00612 |  |  |
| (GeTe)_0.99_(Cu_8_GeSe_6_)_0.01_ | GeTe | a=b=4.153  c=10.674 | Ge:0.0193  Te:0.00725 | 0.123 | 0.962:0.038(0.033) |
|  | Cu_8_GeSe_6_  (*P*6_3_*cm*) | a=b=7.492  c=12.670 | Cu:0.00433  Se:0.00564 |  |  |
| (GeTe)_0.98_(Cu_8_GeSe_6_)_0.02_ | GeTe | a=b=4.138  c=10.629 | Ge:0.0162  Te:0.00739 | 0.131 | 0.953:0.047(0.045) |
|  | Cu_2_Se(*R-*3*m*) | a=b=3.855  c=21.024 | Cu:0.0137  Se:0.0142 |  |  |
| (GeTe)_0.98_(Cu_8_GeSe_6_)_0.02_ | GeTe | a=b=4.138  c=10.629 | Ge:0.0174  Te:0.0076 | 0.119 | 0.927:0.073(0.034) |
|  | Cu_8_GeSe_6_  (*P*6_3_*cm*) | a=b=7.441  c=12.725 | Cu:0.00463  Se:0.00550 |  |  |

Fig. S3. X-ray PDF data for (GeTe)_0.995_(Cu_8_GeSe_6_)_0.005_ (a, d), (GeTe)_0.99_(Cu_8_GeSe_6_)_0.01_ (b, e) and (GeTe)_0.98_(Cu_8_GeSe_6_)_0.02_ (c, f) fitted by the r-GeTe phase/Cu_2_Se (*R-*3*m*) (a, b, c) and r-GeTe phase/Cu_8_GeSe_6_ (*P*6_3_*cm*) (d, e, f) models over the range of 2<*r*<10 Å. The offset is shown by the difference curves (green) below.

Fig. S4. SEM images for (GeTe)_1-_*_x_*(Cu_8_GeSe_6_)*_x_* with *x*=0 (a), *x*=0.005 (b), *x*=0.007 (c), *x*=0.01 (d), *x*=0.02 (e) and *x*=0.04 (f). The corresponding EDS mappings are shown as the inset in (a).


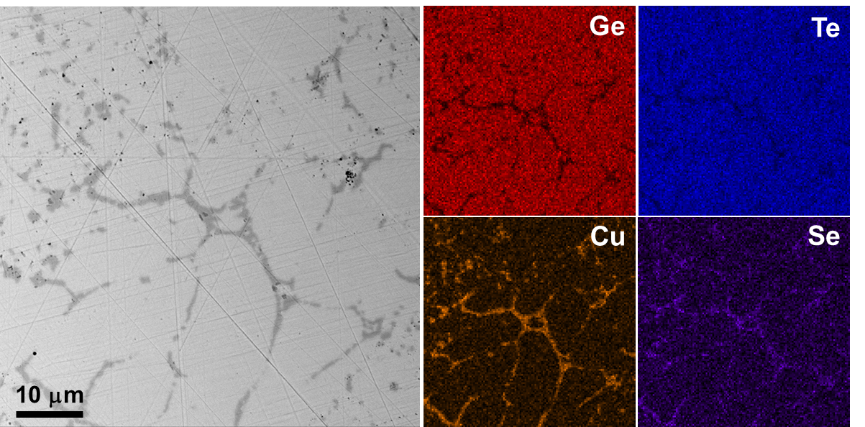


Fig. S5. SEM image and corresponding EDS mappings for (GeTe)_0.98_(Cu_8_GeSe_6_)_0.02_.

Fig. S6. TEM image and corresponding EDS results for (GeTe)_0.993_(Cu_8_GeSe_6_)_0.07_.

Fig. S7. TEM analyses of the (GeTe)_0.98_(Cu_8_GeSe_6_)_0.02_ sample. (a-d) Low magnification TEM image and the corresponding EDS mapping; (e-f) HRTEM image along the [110] direction.

Fig. S8. Heat flow as a function of temperature for (Ge_1-_*_y_*Sb*_y_*Te)_1-_*_x_*(Cu_8_GeSe_6_)*_x_*, indicating a phase transition above 640 K.

Fig. S9. Temperature-dependent Hall carrier concentration (*n*_H_, a), Hall mobility (*μ*_H_, b), resistivity (c), Seebeck coefficient (d), total thermal conductivity (e) and *zT* (f) for (GeTe)_1-_*_x_*(Cu_8_GeSe_6_)*_x_*.

Fig. S10. Temperature-dependent Hall carrier concentration (*n*_H_) and Hall mobility (*μ*_H_) (a), resistivity (b), Seebeck coefficient (c), total thermal conductivity (d), lattice thermal conductivity (e) and *zT* (f) for (Ge_1-_*_y_*Sb*_y_*Te)_0.993_(Cu_8_GeSe_6_)_0.007_.

Fig. S11. Temperature-dependent Hall carrier concentration (*n*_H_) and Hall mobility (*μ*_H_) (a), resistivity (b), Seebeck coefficient (c), total thermal conductivity (d), lattice thermal conductivity (e) and *zT* (f) for (Ge_1-_*_y_*Sb*_y_*Te)_0.98_(Cu_8_GeSe_6_)_0.02_.

Fig. S12. Temperature-dependent Hall carrier concentration (*n*_H_) and Hall mobility (*μ*_H_) (a), resistivity (b), Seebeck coefficient (c), total thermal conductivity (d), lattice thermal conductivity (e) and *zT* (f) for (Ge_1-_*_y_*Sb*_y_*Te)_0.96_(Cu_8_GeSe_6_)_0.04_.

Fig. S13. Vicker hardness for (Ge_1-_*_y_*Sb*_y_*Te)_1-_*_x_*(Cu_8_GeSe_6_)*_x_* alloys (a) with a comparison to that of the literature GeTe-based thermoelectrics (b).

Fig. S14. Schematic and photograph of the setup for measuring the efficiency of a single-leg device.

Fig. S15. Current-dependent output voltage and output power (a) and conversion efficiency (b) under different temperature differences (Δ*T*) for (GeTe)_0.96_(Cu_8_GeSe_6_)_0.04_. Δ*T*-dependent open-circuit voltage and internal resistance for (GeTe)_0.96_(Cu_8_GeSe_6_)_0.04_ (c) and Ge_0.95_Sb_0.05_Te)_0.98_(Cu_8_GeSe_6_)_0.04_ (d).

Fig. S16. Temperature difference (∆*T*)-dependent heat flow for (GeTe)_0.96_(Cu_8_GeSe_6_)_0.04_ (a) and (Ge_0.95_Sb_0.05_Te)_0.98_(Cu_8_GeSe_6_)_0.04_ (b).

**References**

1. G. J. S. Andrew F. May, *Materials Science and Engineering*, **2017**, 11, 1-18.

2. Peter J. Chupas, X. Q, Jonathan C. Hanson, Peter L. Lee, Clare P. Greya, Simon J. L. Billingeb. *Journal of Applied Crystallography* **2003**, 6, 1342-1347.

3. H. A. Lyden, *Phys Rev* **1964**, 134, A1106-A1112.

4. T. Proffen, S. J. L. B., PDFFIT. PDFFIT, *Journal of Applied Crystallography* **1999**, 32, 572-575.

5. C. L. Farrow, P. Juhas, J. W. Liu, *Journal of Physics: Condensed Matter*, **2007**, 19, 335219.

6. J. Kieffer, D. Karkoulis, PyFAI, *Journal of Physics: Conference Series*, **2013**, 425, 202012.

7. P. Juhas, T. Davis, C. L. Farrow, *Journal of Applied Crystallography*, **2013**, 46, 560-566.

8. SJL. Billinge, M. Terban, Songsheng Tao, Long Yang, Y. Rakita, B. Frandsen, *Oxford: Elsevier*, **2023**, 11.

9. Egami, T. Billinge, S. J. L. (2012). Underneath the Bragg Peaks:Structural Analysis of Complex Materials, 2nd ed. *Amsterdam:Elsevier*.

10. M. Liu, X. Zhang, J. Tang, Z. Chen, W. Li and Y. Pei, *Science Bulletin*, **2023**, 68, 2536-2539.
